# Genetic and environmental factors related to the development of myopic maculopathy in Spanish patients

**DOI:** 10.1371/journal.pone.0236071

**Published:** 2020-07-30

**Authors:** Valentina Bilbao-Malavé, Sergio Recalde, Jaione Bezunartea, Maria Hernandez-Sanchez, Jorge González-Zamora, Leyre Maestre-Rellan, José María Ruiz-Moreno, Javier Araiz-Iribarren, Luis Arias, Jorge Ruiz-Medrano, Ignacio Flores-Moreno, Sara Llorente-González, Guillermo Fernández-Sanz, Clara Berrozpe-Villabona, Alvaro Velazquez-Villoria, Ester Carreño, Patricia Fernandez-Robredo, Alfredo Garcia-Layana

**Affiliations:** 1 Ophthalmology Experimental Laboratory, Clínica Universidad de Navarra, Pamplona, Spain; 2 Department of Ophthalmology, Clínica Universidad de Navarra, Pamplona, Spain; 3 Instituto de Investigación Sanitaria de Navarra (IdiSNA), Pamplona, Spain; 4 Red Temática de Investigación Cooperativa en Salud: ‘‘Prevention, Early Detection, and Treatment of the Prevalent Degenerative and Chronic Ocular Pathology” from (RD16/0008/0021), Ministerio de Ciencia, Innovación y Universidades, Instituto de Salud Carlos III, Madrid, Spain; 5 Department of Ophthalmology, Universidad de Castilla-La Mancha, Ciudad Real, Spain; 6 Department of Ophthalmology, Hospital Universitario Puerta de Hierro de Majadahonda, Madrid, Spain; 7 Vissum Corporación Oftalmológica, Alicante, Spain; 8 Instituto Clínico Quirúrgico de Oftalmología, Bilbao, Spain; 9 Department of Ophthalmology, Hospital San Eloy, Bilbao, Spain; 10 Hospital Universitario de Bellvitge, Barcelona, Spain; 11 Department of Ophthalmology, Clínica Universidad de Navarra, Madrid, Spain; 12 Clínica Villoria, Pontevedra, Spain; 13 Hospital Universitario Fundación Jiménez Díaz, Madrid, Spain; University of Florida, UNITED STATES

## Abstract

High myopia and the subsequent degenerative changes of the retina, choroid, and sclera, known as myopic maculopathy (MM), are a serious visual problem in many Asian countries, and are beginning to be so in the south of Europe, especially in the Mediterranean. It is therefore necessary to carry out genetic and environmental studies to determine the possible causes of this disease. This study aims to verify if the genetic factors that have been most related to Asian populations are also associated in two Spanish cohorts. Eight SNPs from six genes (*PAX6*, *SCO2*, *CCDC102B*, *BLID*, *chromosome 15q14*, and *COL8A1*) along with demographic, ophthalmic and environmental factors were analysed in two cohorts from a total of 365 highly myopic subjects and 177 control subjects. The genetic analysis showed that *COL8A1* SNP rs13095226 was associated with the development of choroidal neovascularization (CNV) and also seems to play an important role in the increase of axial length. The SNP rs634990 of *chromosome 15q14* also showed a significant association with MM, although this was lost after the Bonferroni correction. Additional demographic and environmental factors, namely age, sex, smoking status, and pregnancy history, were also found to be associated with MM and CNV in this population.

## Introduction

Myopic maculopathy (MM) is a progressive and complex ophthalmic disease that affects 10% of individuals with high myopia (HM) [1]. MM is the most common cause of vision impairment in these patients, and is also one of the leading causes of legal blindness in developed countries [1]. The prevalence of HM worldwide is increasing at an unprecedented rate; according to the World Health Organization, 2.8% of the world’s population suffered from HM in 2010, and preliminary projections predict that 10% of the population will be affected in 2050 [1]. Theoretically, this increase in the prevalence of HM could eventually lead to an increase in the prevalence of associated pathologic conditions, including MM, which could then increase the incidence of blindness and permanent vision impairment. This will have a significant impact on the public health economy, imposing increased pressure on ophthalmological and low-vision services, as well as on the quality of life and personal development of patients.

MM encompasses a range of degenerative changes of the retina, choroid, and sclera that may develop secondary to mechanical strain caused by eyeball enlargement. The resulting deformation of the posterior pole, known as staphyloma, eventually leads, in most cases, to other conditions such as atrophic, traction, or neovascular lesions [2,3]. Several photographic grading schemes for the classification of MM have been proposed. The most recent of these, published by Ohno-Matsui et al. [4] and Ruiz-Medrano et al. [3], have standardized the definition of MM, and have facilitated comparisons between the findings of epidemiological studies [5–8].

MM is the second most common cause of low vision in Chinese individuals [9,10] and the leading cause of blindness in Japanese individuals [11]. However, to date, the exact mechanism underlying the development of this pathology is not fully understood. It is thought that in addition to environmental factors, genetics may also play an important role in the development of MM. However, how genetics are involved in this process, and whether HM evolves into MM or they are two separate conditions with different underlying causes remain unknown. Knowledge on the genetic background of myopia has expanded dramatically in recent years, particularly since the introduction of genome-wide association studies (GWASs). Several genetic studies, mainly conducted in Asian populations, have identified genes associated with the development of myopia, HM, and MM, namely *SCO2* [12–14], *chromosome 15q14* [15–17], *PAX6* [18–20], *BLID* [14,21], *COL8A1* [22], and *CCDC102B* [23]. Furthermore, the Consortium for Refractive Error and Myopia conducted a GWAS meta-analysis, and identified 161 common variants for refractive error and 9 loci associated with axial length [24].

Although myopia is less prevalent in Caucasians than Asians, MM is also a significant cause of legal blindness and visual impairment in the western hemisphere, especially in the Mediterranean [25–28]. An epidemiologic study showed that the prevalence of MM was significantly higher in a Spanish population than other Caucasian populations [29], and according to the Spanish Blindness Registry is the most common untreatable cause of blindness [30]. As a result, MM is currently a high research priority in Europe, as it has been in Asia for many years. The purpose of the current study was to verify if genetic variants associated with MM in Asian populations are also associated with MM in Spanish individuals.

## Materials and methods

### Study subjects

All procedures carried out in this study conformed to the guidelines of the Declaration of Helsinki. The Institutional Review Board and the Ethics Committee of Clínica Universidad de Navarra (Spain) approved the protocols used in this study. All patients were fully informed of the purpose and procedures, and written consent was obtained from each patient. All cases underwent a detailed ophthalmologic examination including automatic objective refraction, visual acuity assessment, dilated slit-lamp biomicroscopy, axial length measurement by A-scan ultrasound (UD-6000; Tomey, Nagoya, Japan) or partial coherence interferometry (IOLMaster; Carl Zeiss Meditec, Jena, Germany), macular optical coherence tomography (DRI OCT Triton SS-OCT Angio. Topcon, Medical Systems, Inc. Oakland, NJ, USA), and colour fundus photography (Mydiatric Retinal Camera TRC 50 DX, Type IA. Topcon, Medical Systems, Inc. Oakland, NJ, USA). Furthermore, all subjects who agreed to participate in the study were asked to complete a questionnaire about their medical history, smoking habits, and the number of hours spent doing near work and outdoor activities during their childhood.

A total of 365 unrelated HM Spanish Caucasian patients and 177 non-myopic controls were recruited from July 2016 to March 2019. Both, the highly myopic and the control group were made up of two cohorts, one of patients recruited from Clínica Universidad de Navarra (cohort 1) and a second one of patients recruited from various centres of the Red Temática de Investigación Cooperativa OFTARED across Spain (cohort 2). The general inclusion criteria for the study were spherical refractive error ≤-6.00 diopters or axial length ≥26 mm, and age at enrolment >40 years. MM was graded in all fundus photographs of the participant by two trained graders according to the classification systems of Ohno-Matsui et al. and Ruiz-Medrano et al. Any disagreements were resolved by consulting a retinal specialist.

HM patients were divided into two groups depending on the presence or absence of MM. The group without MM included patients without atrophy (category 0 or 1), without traction (category 0) and without neovascularization (category 0). On the other hand, the group with MM included patients with atrophy (category 2, 3 or 4), with or without traction (categories 0 to 5) and with or without neovascularization (category 0, 1, 2a, or 2s). A subanalysis of the MM group was also carried out. For this, patients were classified according to the presence or absence of choroidal neovascularization (CNV). Only patients with CNV due to pathologic myopia were included in the CNV group. Thus, in this group, CNV occurred in eyes with pathologic myopia which was defined as having MM equal to or more serious than diffuse atrophy (category 2) or having a posterior staphyloma. To avoid the inclusion of CNV occurring in highly myopic eyes, probably due to other causes, the following exclusion criteria were established: patients with inflammatory CNV such as those related to punctate inner choroidopathy (PIC), idiopathic CNV, any evidence of age-related macular degeneration (AMD) such as retinal drusen, angioid streaks, presumed ocular histoplasmosis syndrome or lacquer cracks due to trauma. Other exclusion criteria were patients with known genetic diseases associated with myopia, such as Stickler or Marfan syndrome and any type of ocular media opacity precluding visualization of the fundus.

### Genotyping

Genomic DNA was extracted from oral swabs using QIAcube (Qiagen, Hilden, Germany) and processed in the Ophthalmology Experimental Laboratory of the Clínica Universidad de Navarra (Spain). A set of eight SNPs of six previously identified MM-associated genes in Asian populations (*COL8A1*, *SCO2*, *CCDC102B*, *chromosome 15q14*, *PAX6*, and *BLID*) were genotyped by an ABI Prism 7300 Real-Time PCR System (Life Technologies, Carlsbad, CA, USA) using validated TaqMan assays (Applied Biosystems, Foster City, CA, USA) for each gene: *COL8A1* (rs669676/C_8192922_10 and rs13095226/C_26159211_10), *SCO2* (rs74315510/C_27532167_10 and rs8139305), *CCDC102B* (rs11873439/C_32010914_30), *chromosome 15q14* (rs634990/C_2088259_10), *PAX6* (rs644242/C_898191_10), and *BLID* (rs577948/C_3150597_10). None of the SNPs were in linkage disequilibrium.

### Expression

Relative quantification analysis was conducted to confirm the expression of *CCDC102B* (Hs00227117_m1), *COL8A1* (Hs00156669_g1), and *SCO2* (Hs00192979_m1) in human eye tissues from Spanish donors using Taqman expression assays. This analysis was performed in short, postmortem eye bulbs (the retina was obtained from six donor eyes, the retinal pigment epithelium (RPE) from three donor eyes, and the sclera from two donor eyes) provided by the anatomy department of the Universidad de Navarra (Spain). The mRNA was extracted from specific tissues (sclera, retina and RPE) with the ABI PRISM^™^ 6100 Nucleic Acid PrepStation (Life Technologies, Carlsbad, CA, USA). Using the qScript cDNA Supermix Kit (Quanta Biosciences, Gaithersburg, MD, USA), 1000 ng of each mRNA was reverse transcribed using a 2720 Thermal Cycler (Life Technologies, Carlsbad, CA, USA). The 7300 Real-Time PCR System (Life Technologies, Carlsbad, CA, USA) was used for amplification, and two housekeeping genes (glyceraldehyde 3-phosphate dehydrogenase and ß-actin) were used as internal controls. The expression of these genes was determined in the sclera and RPE, and compared to the expression in the retina.

### Statistical analyses

General characteristics were compared between the groups using the Student´s t-test for continuous variables (age, refractive error, and axial length) or the chi-square test for categorical variables (sex, arterial hypertension, hypercholesterolemia, and smoking history). The frequencies of alleles and genotypes were calculated in all the groups and were compared using the chi-square test and Fisher’s exact test, and corresponding odds ratios (ORs) were calculated. All SNPs analysed in this study were in Hardy-Weinberg equilibrium.

Univariable logistic regression adjusted for sex, age and smoking history was used to estimate ORs and 95% CIs using SNPStats software [31] (Institut Català d’Oncologia, Barcelona, Spain). Analyses were performed for each genetic variant independent of other variants using codominant, dominant, recessive, and/or overdominant genetic models. Akaike´s information criterion was then used to choose the inheritance model that best fit the data. The Bonferroni method was used to correct for multiple comparisons. All statistical analyses were conducted with SPSS 20.1 Software (SPSS Inc., Chicago, IL, USA). For all statistical tests, corrected p values < 0.05 (two-tailed) were considered statistically significant.

## Results

### Demographic characteristics

The global demographic characteristics of the study population are shown in Table 1. From HM group, cohort 1 and 2, include 231 and 134 patients respectively. With respect of the control group, cohort 1 include 124 patients and cohort 2 include 53 patients. A total of 542 participants, including 365 highly myopic subjects and 177 control subjects were enrolled.

**Table 1:** Demographic characteristics of the study population, including cohort 1 and 2 from the HM and control groups.

	High Myopia (HM)																		Control Group				
	HM with Myopic Maculopathy				HM without Myopic Maculopathy				p-value 1	HM with CNV				HM without CNV				p-value 2	No HM				p-value 3
	Cohort1	Cohort 2	p-value	Total	Cohort 1	Cohort 2	p-value	Total		Cohort 1	Cohort 2	p-value	Total	Cohort 1	Cohort 2	p-value	Total		Cohort 1	Cohort 2	p-value	Total	
Number of Patients	176	103		**279**	55	31		**86**		98	79		**177**	133	55		**188**		124	53		**177**	
Age (Mean ± SD)	66.65±12.4	57.6 ±14.3	0.08	**63.40 (±13.85)**	57.60 ±9.1	53.16 ±12.4	0.05	**53.97 (±13.01)**	***4*.*1x10***^***-5***^	67.98 ±13.0	60.2 ±13.3	***0*.*002***	**64.23 (±14.23)**	61.89 ±11.1	53.15 ±11.8	***6*.*2x10***^***-4***^	**58.58 (±13.71)**	***9*.*3x10***^***-4***^	76.60 ±14.8	71.15 ±13.7	0.120	**73.17 (±6.82)**	***2*.*1x10***^***-5***^
Female Gender (%)	118 (67.0)	67 (65.0)	0.880	**185 (66.3%)**	30 (54.4)	17 (54.8)	0.990	**47 (54.70%)**	0.0547	68 (69.4)	49 (62.0)	0.33	**117 (66.1%)**	79 (59.4)	36 (65.4)	0.530	**115 (61.17%)**	0.3310	65 (52.4)	32 (59.4)	0.410	**97 (53.6%)**	***0*.*0329***
Refractive Error (Diopters ± SD)	-13.80 ±4.69	-14.09 ±4.69	0.345	**-13.90 (±4.28)**	-10.20 ±3.25	-9.22 ±2.40	0.220	**-9.53 (±2.39)**	***3*.*7x10***^***-4***^	-14.15 ±4.99	-13.42 ±4.10	0.340	**-13.77 (±4.59)**	-12.12 ±4.19	-12.22 ±4.47	0.695	**-12.10 (±4.26)**	***0*.*0004***	-1.61 ±0.94	-1.82 ±0.90	0.780	**-1.77 (±0.93)**	***4*.*1x10***^***-7***^
Axial Length (mm ± SD)	30.63±2.61	30.3 ±2.40	0.855	**30.49 (±2.454)**	27.80 ±1.99	27.20 ±1.89	0.440	**27.38 (±1.15)**	***9*.*7x10***^***-4***^	30.06 ±2.51	30.4 ±2.62	0.780	**30.25 (±2.51)**	29.70 ±2.84	29.20 ±2.24	0.690	**29.61 (±2.70)**	***0*.*009***	23.65 ±0.56	23.20 ±0.42	0.560	**23.46 (±0.49)**	***1*.*1x10***^***-7***^
Tobacco Smokers (%)	69 (39.2)	31 (32.6)	0.315	**100 (35.8%)**	31 (56.3)	10 (37.8)	0.150	**41 (50%)**	***0*.*0399***	35 (35.7)	24 (32.0)	0.630	**59 (34.1%)**	64 (48.1)	17 (36.1)	0.190	**81 (43.08%)**	***0*.*0434***	29 (23.3)	14 (26.4)	0.320	**43 (23.7%)**	***0*.*0002***
Hypertension (%)	56 (31.8)	32 (33.6)	0.690	**88 (31.5%)**	16 (29.1)	4 (14.8)	0.180	**20 (24.3%)**	0.2203	33 (33.7)	27 (36.0)	0.870	**60 (34.7%)**	39 (29.3)	9 (19.1)	0.200	**48 (25.5%)**	0.3320	62 (50.0)	23 (43.4)	0.210	**85 (46.97%)**	***0*.*0001***
Hypercholesterolemia (%)	57 (32.3)	21 (22.6)	0.11	**78 (27.9%)**	21 (38.2)	4 (14.8)	***0*.*040***	**25 (30.5%)**	0.7790	38 (38.8)	17 (23.2)	***0*.*040***	**55 (32.2%)**	40 (30.0)	8 (17.0)	0.190	**48 (25.5%)**	0.2240	42 (33.8)	16 (30.1)	0.880	**58 (32.05%)**	0.6930

HM: High myopia; CNV: Choroidal neovascularization; SD: Standard deviation.

^1)^ P-value comparing HM-No MM with HM-MM.

^2)^ P-value comparing HM-CNV+ with HM-No CNV

^3)^ P-value comparing HM with Control group

Significance p < 0.05

Of the 365 highly myopic patients, 279 had MM and 86 did not have any degenerative changes specific to pathologic myopia. Of the group of MM patients, 177 (63.4%) had CNV. The mean age of the participants was 61.19 (40–94) years. The subjects from the group with MM and CNV were significantly older than those without MM (p = 4.1x10^-5^) and CNV (p = 9.3x10^-4^). In this sense, the comparative results between cohort 1 and cohort 2 showed significant differences between the group with (p = 0.002) and without CNV (p = 6.2x10^-4^).The control subjects were also older than the highly myopic patients with a statistically significant difference (p = 2.1x10^-5^).

When sex was analysed, the results showed that HM was more common among women with a statistically significant difference (p = 0.0329). Women also tended to develop more advanced stages of MM and CNV, though these differences were not statistically significant (Table 1).

### Ophthalmic characteristics

**Fig 1:**
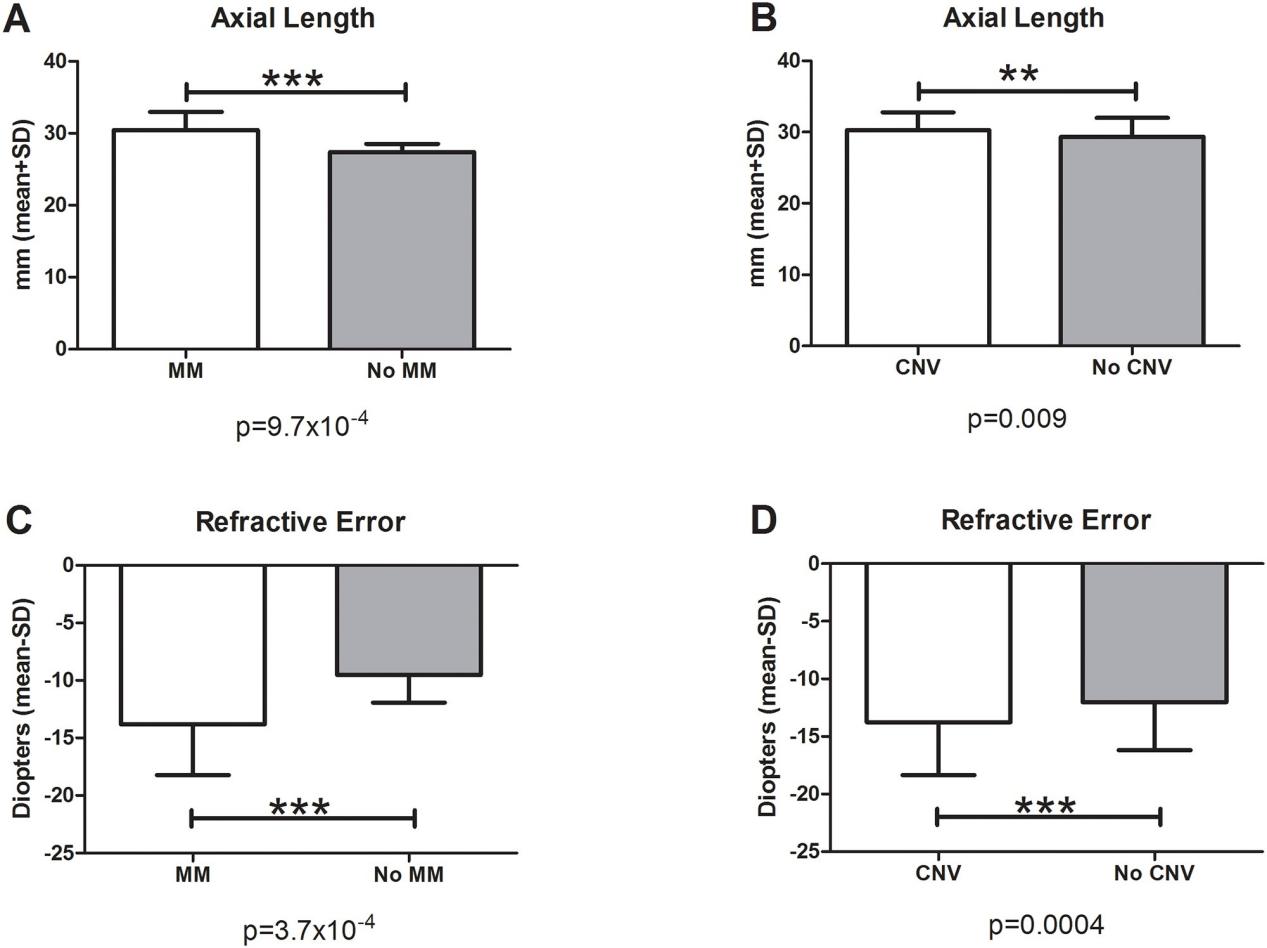
Axial length and refractive error. In the groups with MM (A) and CNV (B), the axial length was significantly increased compared to the groups without MM (A) and without CNV (B). Analysis of refractive error showed that subjects with MM (C) and CNV (D) had a significantly higher degree of myopia than subjects without MM (C) and without CNV (D).

Axial length was significantly increased in patients with HM compared to the control group (p = 1.1x10^-7^) and also in patients with MM compared to those without MM (p = 9.7x10^-4^) (Fig 1A). Axial length was also significantly different between patients with CNV and patients without CNV (p = 0.009) (Fig 1B).

Analysis of refractive error showed that subjects with HM and MM had a higher degree of myopia than controls (p = 4.1x10^-7^) and subjects without MM (p = 3.7x10^-4^). Again, refractive error was also significantly different between patients with CNV and patients without CNV (p = 0.0004) (Fig 1C and 1D). Cohort 1 and 2 do not show differences with respect of axial length and refractive error (Table 1).

### Environmental characteristics

**Fig 2:**
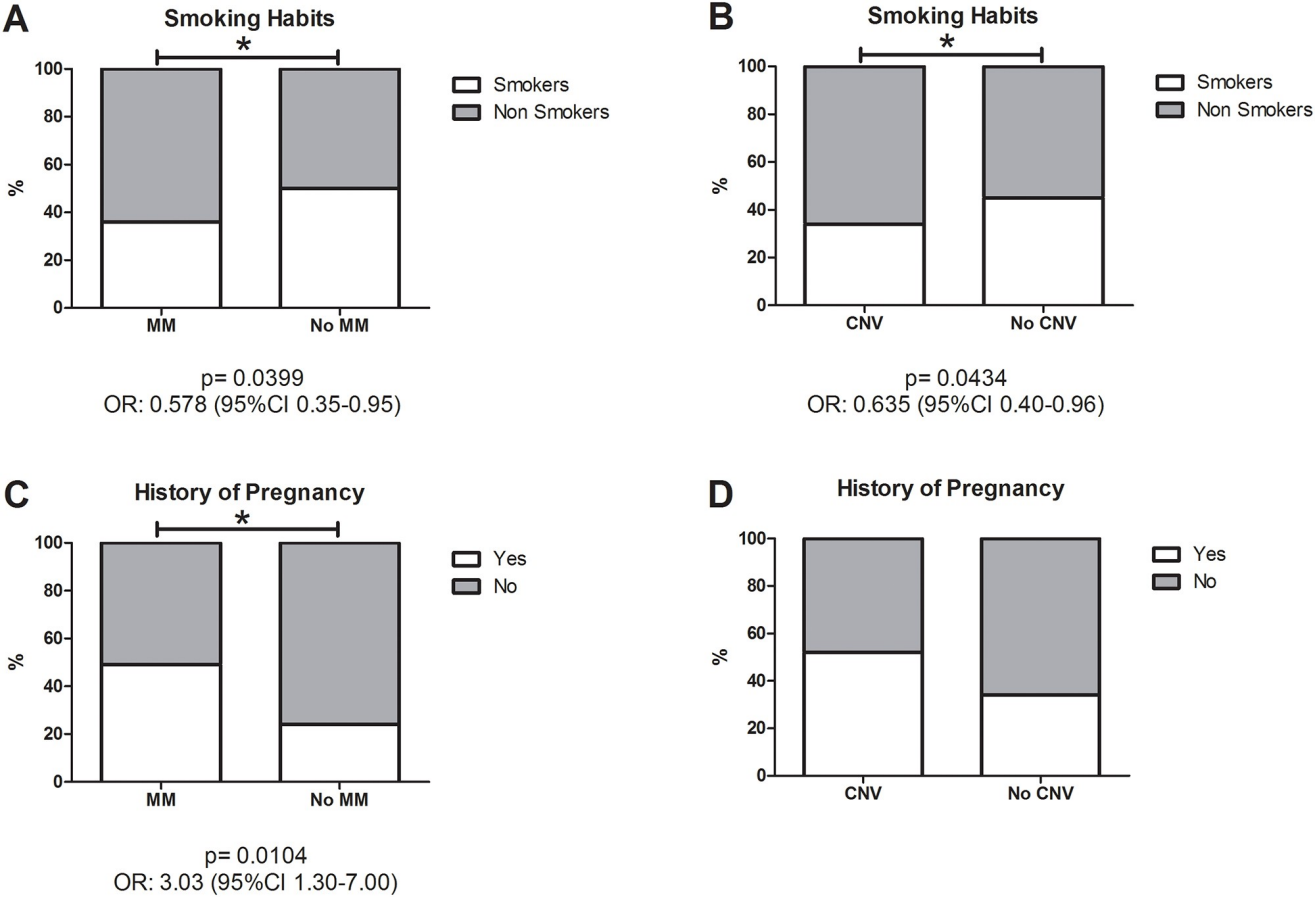
Smoking habit and history of pregnancy. A significantly higher number of non-smokers individuals were found in the MM (A) and CNV groups (B) compared to the groups without MM (A) and without CNV (B). A significantly greater number of woman who had been pregnant at least once were found in the MM group compared to the group without MM (C), but no statistically significant differences were found when comparing the groups with and without CNV (D).

When smoking status was analysed, a significantly higher number of smokers were found in the HM group compared with the control group (p = 0.0002) (Table 1). Nevertheless, non-smokers were found to be significantly more likely to develop MM (p = 0.0399; odds ratio (OR) 0.578 (95% confidence interval (95%CI) 0.35–0.95) and CNV (p = 0.0434; OR 0.635 (95%CI 0.40–0.96)) (Fig 2A and 2B). Among the women included in this study, 39.8% had been pregnant at least once, and these women were significantly more likely to develop MM (p = 0,0104; OR 3.03 (95%CI 1.30–7.00)) but no CNV (Fig 2C and 2D). Although women who had taken hormone replacement therapy tended to develop more severe stages of MM, this difference was not statistically significant.

**Fig 3:**
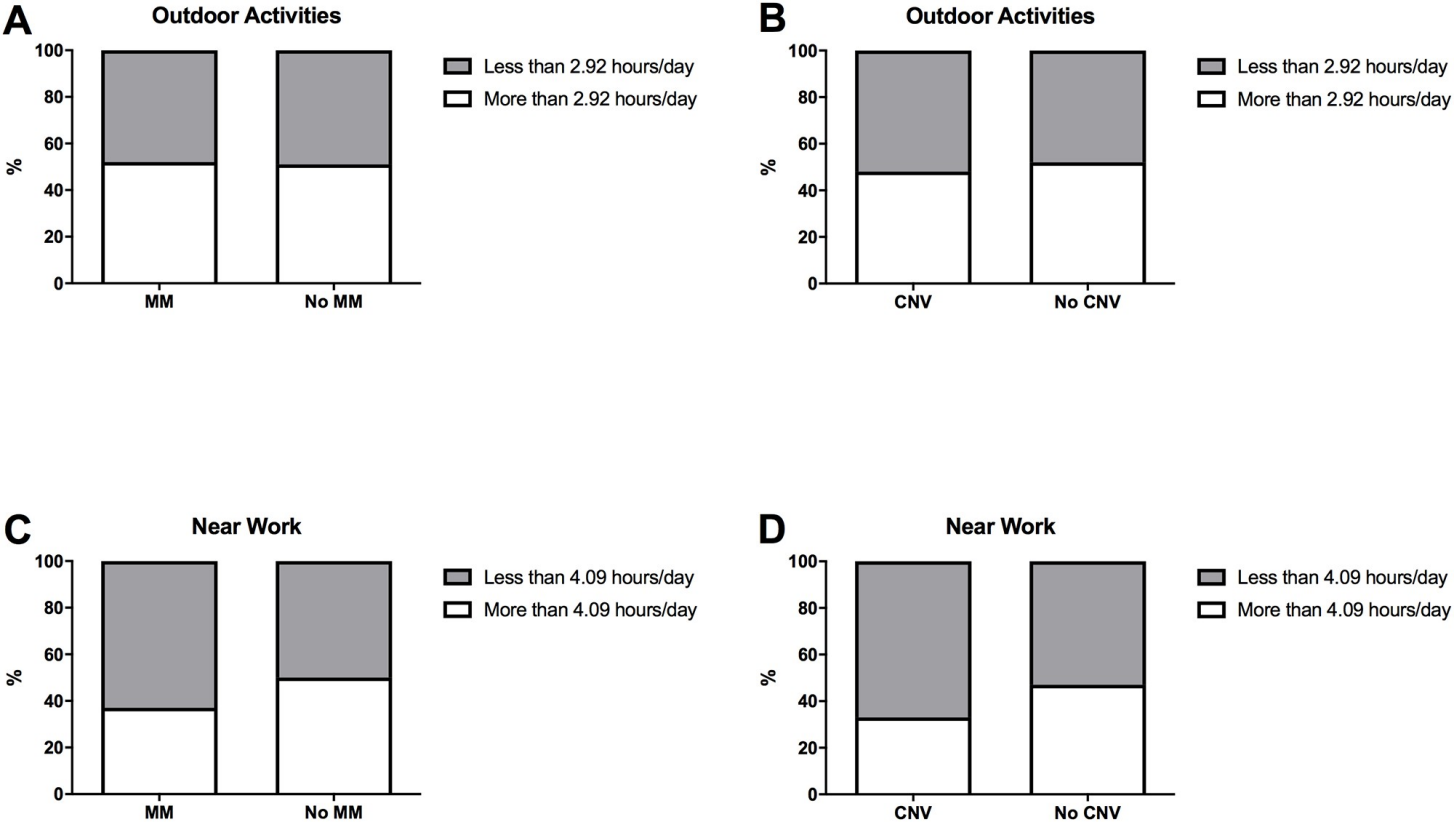
Outdoor activities and near work in childhood. We calculate the mean number of hours spent doing outdoor activities and near work during childhood, and analyse the proportion of subjects that were above and below that number. No statistically significant differences were found when comparing the groups with and without MM and with and without CNV (A-D).

No significant differences were found with respect to the time spent doing outdoor activities and near work during childhood between the groups with and without MM and with and without CNV (Fig 3). On the other hand, hypertension was significantly more common in the HM group (p = 0.0001), but there were no differences in the incidence of hypertension or hypercholesterolemia between the groups with and without MM and CNV. Hypercholesterolemia was significantly more common in cohort 1 subjects from the group without MM (p = 0.040) and with CNV (p = 0.040) (Table 1).

### Allele and genotype frequencies

All SNPs analysed were in Hardy-Weinberg equilibrium (Table 2). Tables 3, 4 and 5 show the genotype frequencies in the subjects with and without HM, MM and CNV, respectively. Minor allele frequencies are shown in Table 2. No statistically significant differences were found in the allele frequency of any of the eight SNPs analysed between the subjects with and without HM, MM and CNV. However, *COL8A1* SNPs (rs13095226 and rs669676) showed a certain tendency to be more frequent in patients with HM than in controls. The SNP rs634990 of *chromosome 15q14* also tended to be more frequent in the MM group without any significance (Table 2).

**Table 2:** Table of Minor Allele Frequencies (MAF) in different study groups.

		High Myopia (HM)															Control Group					
		HM with Myopic Maculopathy			HM without Myopic Maculopathy			p-value 1	HM with CNV			HM without CNV			p-value 2		No HM			p-value 3	HW Equili.
	Minor Allele	MAF Cohort1	MAF Cohort 2	MAF Total	MAF Cohort 1	MAF Cohort 2	MAF Total		MAF Cohort 1	MAF Cohort 2	MAF Total	MAF Cohort 1	MAF Cohort 2	MAF Total		MAF HM	MAF Cohort 1	MAF Cohort 2	MAF Total		
**rs11873429**	C	0.03	0.03	**0.03**	0.04	0.0	**0.03**	**0.94**	0.04	0.02	**0.03**	0.03	0.04	**0.03**	**0.93**	**0.03**	0.04	0.03	**0.04**	**0.91**	0.33
**rs577948**	G	0.46	0.44	**0.45**	0.39	0.48	**0.42**	**0.62**	0.47	0.44	**0.46**	0.42	0.46	**0.43**	**0.39**	**0.44**	0.42	0.56	**0.46**	**0.61**	0.44
**rs669676**	G	0.53	0.54	**0.54**	0.43	0.52	**0.46**	**0.11**	0.54	0.46	**0.5**	0.49	0.46	**0.46**	**0.26**	**0.48**	0.42	**0.38**	**0.41**	**0.07**	0.17
**rs13095226**	C	0.10	0.16	**0.12**	0.05	0.12	**0.08**	**0.10**	0.11	0.18	**0.14**	0.08	0.11	**0.09**	**0.063**	**0.11**	0.08	**0.03**	**0.07**	**0.065**	0.19
**rS644242**	A	0.04	0.05	**0.04**	0.05	0.06	**0.05**	**0.75**	0.04	0.04	**0.04**	0.04	0.07	**0.05**	**0.81**	**0.04**	0.07	**0.05**	**0.06**	**0.27**	0.58
**rs634990**	T	0.47	0.44	**0.46**	0.41	0.32	**0.38**	**0.09**	0.48	0.43	**0.46**	0.44	0.39	**0.43**	**0.48**	**0.44**	0.47	0.60	**0.50**	**0.13**	0.13
**rs74315511**	T	0	0	**0**	0	0	**0**	**-**	0	0	**0**	0	0	**0**	**-**	**0**	0	0	**0**	**-**	-
**rs8139305**	G	0	0	**0**	0	0	**0**	**-**	0	0	**0**	0	**0**	**0**	**-**	**0**	0	0	**0**	**-**	-

HM: High myopia; CNV: Choroidal neovascularization; SD: Standard deviation.

^1)^ P-value comparing HM-No MM with HM-MM.

^2)^ P-value comparing HM-CNV+ with HM-No CNV

^3)^ P-value comparing HM with Control group

Significance p < 0.05

**Table 3:** Comparison of the genotype frequencies between the HM vs. No HM group.

		Cohort 1			Cohort 2						
SNPs	Genotype	Control Genotype frec (%) Genotype cases (n)	HM Genotype frec (%) Genotype cases (n)	Genot. Uncor. p-value	Control Genotype frec (%) Genotype cases (n)	HM Genotype frec (%) Genotype cases (n)	Genot. Uncor. p-value	Control Genotype frec (%) Genotype cases (n)	HM Genotype frec (%) Genotype cases (n)	Genotype Uncorrected p-value	Genotype OR (95% CI)
**rs11873429**	AA/AC/CC	91.3/8.7/0 63/3/0	93.4/6.0/0.6 157/10/1	0.79	94.6/5.4/0 35/2/0	95.2/4.8/0 79/4/0	0.76	92.5/7.5/0 98/8/0	94/5.6/0.4 235/14/1	C; 0.63	0.80 (0.3–2.1)
**rs577948**	AA/AG/GG	34.4/48.7/17.2 22/31/11	29.8/51.6/18.6 48/83/30	0.71	26.9/34.6/38.5 7/9/10	33.3/43.2/23.5 27/35/19	0.53	32.2/44.4/23.3 29/40/21	31/48.8/20.2 75/118/49	C; 0.62	1.24 (0.7–2.3)
**rs669676**	AA/AG/GG	35.4/45.6/19 28/36/15	29.3/44.2/26.5 63/95/57	0.27	33.3/56.7/10 10/17/3	30.7/46.5/22.8 31/47/23	0.22	34.9/48.6/16.5 38/53/18	29.3/45.2/25.5 92/142/80	R; 0.08	1.65 (0.9–3.0)
**rs13095226**	CC/CT/TT	83.8/16.2/0 62/12/0	82.3/15.8/1.9 177/34/4	0.24	93.5/6.5/0 29/2/0	73/23.4/3.6 81/26/4	0.13	86.7/13.3/0 91/14/0	79.3/18.6/2.1 256/60/7	R; 0.07	NA (0.0-NA)
**rS644242**	AA/AC/CC	87.6/12.4/0 78/11/0	93.5/5.6/0.9 101/6/1	0.37	90/10/0 27/3/0	90.1/9.9/0 100/11/0	0.6	88.2/11.8/0 105/14/0	91.8/7.8/0.4 201/17/1	C; 0.64	0.68 (0.3–1.6)
**rs634990**	CC/CT/TT	30.3/46.1/23.6 27/41/21	29.5/49.3/21.2 64/107/46	0.40	13.3/53.3/33.3 4/16/10	39.6/37.8/22.5 44/42/25	***0*.*017***	26.1/47.9/26.1 31/57/31	33.1/45.4/21.5 108/148/70	D; 0.43	0.71 (0.4–1.2)
**rs74315511**	CC/CT/TT	89	213	-	30	110	-	119	322	-	-
**rs8139305**	AA/AG/GG	88	213	-	29	110	-	117	322	-	-

Chr: Chromosome; Genotype freq: Genotype frequency; OR: Odds ratio; CI: Confidence interval; P-value: value from logistic regression model; P value significance < 0.05. C; Codominant model (XX vs XY vs YY), R; Recessive model (XX-XY vs YY) D; Dominant model (XX vs XY-YY).

**Table 4:** Comparison of the genotype frequencies between the MM vs. No MM group.

		Cohort 1			Cohort 2						
SNPs	Genotype	MM-Genotype frec (%) Genotype cases (n)	MM+ Genotype frec (%) Genotype cases (n)	Genot. Uncor. p-value	MM- Genotype frec (%) Genotype cases (n)	MM+ Genotype frec (%) Genotype cases (n)	Genot. Uncor. p-value	MM- Genotype frec (%) Genotype cases (n)	MM+ Genotype frec (%) Genotype cases (n)	Genotype Uncorrected p-value	Genotype OR (95% CI)
**rs11873429**	AA/AC/CC	94.9/2.6/2.6 37/1/1	93/7.0/0 119/9/0	0.08	100/0/0 15/0/0	94.1/5.9/0 64/4/0	0.24	96.3/1.8/1.8 52/1/1	93.4/6.6/0 183/13/0	C; 0.076	3.86 (0.5–38.4)
**rs577948**	AA/AG/GG	42.2/36.8/21 16/14/8	26/56.1/17.9 32/69/22	0.19	25/55/20 5/11/4	36/39.3/24.7 22/24/15	0.38	36.2/43.1/20.7 21/25/12	29.4/50.5/20.1 54/93/37	C; 0.49	1.26 (0.7–2.5)
**rs669676**	AA/AG/GG	27.4/31.4/41.2 14/16/21	29/48.8/22.2 47/79/36	***0*.*006***	21.7/60.9/17.4 5/14/4	33.3/42.3/24.4 26/33/19	0.37	25.7/40.5/33.8 19/30/25	30.4/46.7/22.9 73/112/55	R; 0.06	0.54 (0.3–1.1)
**rs13095226**	CC/CT/TT	89.4/10.6/0 42/5/0	80.6/17.6/1.8 133/29/3	0.29	76.9/23.1/0 20/6/0	71.8/23.5/4.7 61/20/4	0.44	84.9/15.1/0 62/11/0	77.6/19.6/2.8 194/49/7	R; 0.10	NA (0.00-NA)
**rS644242**	AA/AC/CC	90.9/9.1/0 10/1/0	94.6/5.4/0 88/5/0	0.88	88.4/11.6/0 23/3/0	90.6/9.4/0 77/8/0	0.86	89.1/10.8/0 33/4/0	92.3/7.2/0.5 168/13/1	C; 0.83	0.84 (0.2–3.1)
**rs634990**	CC/CT/TT	36.7/44.9/18.4 18/22/9	27.7/50.6/21.7 46/84/36	0.11	48/40/12 12/10/3	37.2/37.2/25.6 32/32/22	0.15	40.5/43.2/16.2 30/32/12	31/46/23 78/116/58	***R; 0*.*043*** *0.270	2.08 (1.0–4.4)
**rs74315511**	CC/CT/TT	49	164	-	26	83	-	75	247	-	-
**rs8139305**	AA/AG/GG	47	164	-	26	82	-	77	246	-	-

Chr: Chromosome; Genotype freq: Genotype frequency; OR: Odds ratio; CI: Confidence interval; P-value: value from logistic regression model adjusted by age and gender; P value significance < 0.05. C; Codominant model (XX vs XY vs YY), R; Recessive model (XX-XY vs YY) D; Dominant model (XX vs XY-YY).

* Bonferroni correction p value.

**Table 5:** Comparison of the genotype frequencies between CNV vs. No CNV group.

		Cohort 1			Cohort 2						
SNPs	Genotype	CNV-Genotype frec (%) Genotype cases (n)	CNV+ Genotype frec (%) Genotype cases (n)	Genot. Uncor. p-value	CNV-Genotype frec (%) Genotype cases (n)	CNV+ Genotype frec (%) Genotype cases (n)	Genot. Uncor. p-value	CNV-Genotype frec (%) Genotype cases (n)	CNV+ Genotype frec (%) Genotype cases (n)	Genotype Uncorrected p-value	Genotype OR (95% CI)
**rs11873429**	AA/AC/CC	94.4/4.5/1.1 84/4/1	92.3/7.7/0 72/6/0	0.33	92.9/7.1/0 26/2/0	96.4/3.6/0 53/2/0	0.35	94/5.1/0.9 110/6/1	94/6/0 125/8/0	C; 0.49	1.69 (0.4–3.6)
**rs577948**	AA/AG/GG	33.3/48.9/17.8 30/44/16	25.4/54.9/19.7 18/39/14	0.69	27.8/52.8/19.4 10/19/7	37.8/35.6/26.6 17/16/12	0.28	31.7/50/18.3 40/63/23	30.2/47.4/22.4 35/55/26	C; 0.28	01.34 (0.6–2.9)
**rs669676**	AA/AG/GG	26.4/44.7/28.9 32/54/35	31.9/45/23.1 29/41/21	0.40	26.2/54.8/19.1 11/23/8	33.9/40.7/25.4 20/24/15	0.43	26.2/47/26.8 43/77/44	32.7/43.3/24 49/65/36	R; 0.69	0.9 (0.5–1.5)
**rs13095226**	CC/CT/TT	83.6/16.4/0 102/20/0	81.5/15.2/3.3 75/14/3	***0*.*032***	77.3/22.7/0 34/10/0	70.2/23.9/6 47/16/4	0.07	81.9/18.1/0 136/30/0	76.7/18.9/4.4 122/30/7	*R; 0*.0023*0.*013*	***NA (0*.*00-NA)***
**rS644242**	AA/AC/CC	94.9/5.1/0 37/2/0	92.6/5.9/1.5 63/14/1	0.70	86.7/13.3/0 39/6/0	92.5/7.5/0 62/5/0	0.29	90.5/9.5/0 76/8/0	86.2/13.1/0.7 125/19/1	C; 0.51	NA (0.00-NA)
**rs634990**	CC/CT/TT	34.7/43/22.3 42/52/27	23.6/57/19.4 22/53/18	0.33	44.4/33.3/22.2 20/15/10	37.3/40.3/22.4 25/27/15	0.91	37.3/40.4/22.3 62/67/37	29.4/50/20.6 47/80/33	D; 0.28	1.26 (0.8–2.1)
**rs74315511**	CC/CT/TT	120	92	-	44	66	-	164	158	-	-
**rs8139305**	AA/AG/GG	120	93	-	44	66	-	164	159	-	-

Chr: Chromosome; Genotype freq: Genotype frequency; OR: Odds ratio; CI: Confidence interval; P-value: value from logistic regression model adjusted by age and gender; P value significance < 0.05. C; Codominant model (XX vs XY vs YY), R; Recessive model (XX-XY vs YY) D; Dominant model (XX vs XY-YY).

* Bonferroni correction p value.

In the genotype frequencies analysis between HM and no HM the SNP rs634990 of *chromosome 15q14* exhibited a significant association (p = 0.017) with HM in cohort 2, however this association was lost when both cohorts were together (Table 3). Likewise, this SNP was the only one to show a significant difference in genotype frequency between subjects with and without MM (p = 0.043). In the recessive model (CC/CT vs TT), the TT genotype appeared to be significantly more frequent in patients with MM than in those without MM (OR 2.08; (95% CI 1.0–4.4)), but this significance was lost after the Bonferroni correction (p = 0.270) (Table 4).

The GG genotype of the SNP rs669676 of *COL8A1* was significantly more frequent in patients without MM (p = 0.006) in cohort 1. When both cohorts were together, this association was lost but the recessive model (AA/AG vs GG) showed a certain tendency to be more frequent in patients without MM (Table 4).

With respect to the genotype frequencies of the eight SNPs among the MM patients with and without CNV, *COL8A1* SNP rs13095226 showed significant differences in genotype frequency in patients with CNV (p = 0.032) in cohort 1 and a tendency in cohort 2 (p = 0.07). When both cohorts were together, in the recessive model (TT/CT vs. CC), the TT/CT genotypes of this SNP were also significantly more frequent in patients with CNV (p = 0.0023) and continued to be significant after the Bonferroni correction (p = 0.013) (Table 5).

Likewise, the C and G alleles of the *SCO2* SNPs rs74315511 and rs8139305, respectively, were not detected in any of the genotyped patients, suggesting that the frequencies of these alleles in Spanish individuals are very low. Therefore, in this study, these SNPs were monomorphic polymorphisms (Tables 3, 4 and 5).

### Gene expression in human eye tissue

**Fig 4:**
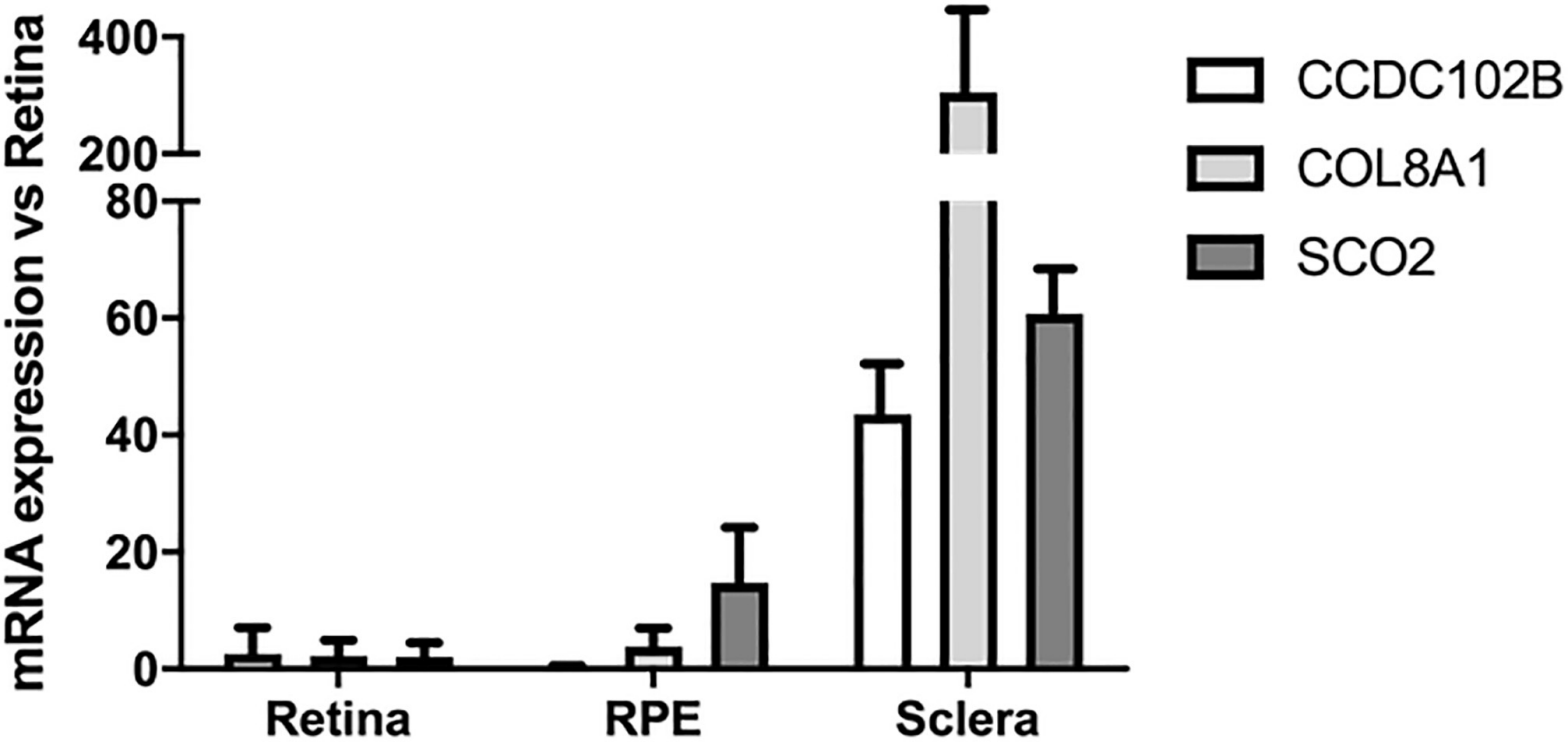
Human eye tissues expression. CCDC102B, COL8A1 and SCO2 genes were expressed in retina, RPE and sclera of cadaveric donors. COL8A1 showed an overexpression of more than 300 times in human sclera when compared with the expression in RPE and retina.

To investigate the expression of *CCDC102B*, *COL8A1*, and *SCO2* in the eye tissue of Spanish individuals, the expression of these genes was evaluated in the sclera and RPE of Spanish cadaveric donors. To obtain reference values and conduct comparisons, the expression of these genes in the retina was evaluated. According to this, the expression of *COL8A1* was 300 times greater in the sclera than in the retina. All three genes were expressed in the eye tissue analysed in this study (Fig 4), but only *COL8A1* was associated with the development of CNV in MM.

## Discussion

MM is a multifactorial and highly complex disease responsible for vision impairment and blindness. Currently, it is not possible to predict which eyes will develop this disease, as a result, recent studies have focused on the use of genetics to detect patients at a higher risk. However, all genetic loci discovered to date indicate that refractive development is a heterogeneous process mediated by a number of overlapping biological processes. Therefore, it is unlikely that there is a single gene (or family of genes) that is solely responsible for the development of chorioretinal atrophy or neovascular lesions [32].

This study aimed to verify if some SNPs associated with HM and MM in Asian populations are also associated with these conditions in Spanish individuals. According to the results obtained, a weak relationship between the studied SNPs and MM and CNV was identified in this European cohorts. *COL8A1* was significantly associated with the development of CNV, as previously reported by our group [22]. In addition, *chromosome 15q14* was significantly associated with the development of MM, although this significance was lost when the Bonferroni correction was applied. Furthermore, significant relationships were identified between some environmental and ophthalmic factors and MM and CNV in this population. The study was carried out in two Spanish cohorts obtaining similar results, what allows to validate the genetic and epidemiological results.

Axial length was significantly increased in patients with HM, MM and CNV compared to their controls. This suggests that increased axial length is a risk factor for the development of pathologic alterations in HM patients [33,34], as shown in previous studies [21,35]. With respect to refractive error, it is thought that subjects with MM and increased axial length have a higher degree of myopia [34,35]. Correlating with this, in this study, significant differences in refractive error were identified when comparing the groups with and without HM, MM and CNV.

In recent years, environmental factors have been implicated in the development of MM [1]. In the current study, associations between environmental and demographic factors and MM and CNV development were investigated, and statistically significant differences were identified with respect to age, sex, smoking status, and pregnancy history.

With regards to age, we found that the mean age of patients with MM and CNV was significantly higher than that of patients without MM and CNV, respectively. These findings correlate with previous reports that show that increased age, and thus retinal thinning, is an important risk factor for the development of pathologic changes in HM patients [33].

Analysis of sex showed that HM was more common among women, and that they had a greater tendency to develop more advanced stages of MM, including CNV, though these differences were not significant. It has been hypothesized that this could be due to the role of oestrogen and 17β-oestradiol in ocular angiogenesis [36]. When we analysed the pregnancy history and hormone replacement therapy status of the recruited women, we observed that women who had been pregnant were more likely to develop complications of myopia like MM. The physiological corneal modifications and hormonal changes that occur in pregnancy may increase myopia [37,38]. Nevertheless, this is believe to be temporary, and pregnancy has not been clearly identified as a risk factor, indeed, a number of studies have found no association between the two [38,39]. Despite this, in this study, a positive association was noted between pregnancy and MM, and women who had taken hormone replacement therapy also tended to develop more advanced stages of MM. Therefore, further investigation is required to elucidate the contribution of these factors to the development of MM.

With respect to smoking status, the proportion of non-smokers was significantly higher in subjects with MM and CNV than in those without MM and CNV, respectively. An experimental study undertaken in chicks showed that nicotinic antagonists inhibit experimental myopia, so it is thought that nicotinic receptors may play a role in ocular development. Nicotine, one of the many components of cigarette smoke, induces a paradoxical upregulation of nicotinic receptors along with rapid receptor desensitization, sometimes causing agonists to behave as time-averaged antagonists in many biological systems [40]. This may explain the results of previous studies that found parental smoking to be associated with lower myopic prevalence and more hyperopic refractions [41,42]. However, the relationship between tobacco and myopia is controversial, and other studies have found no significant association between the two [43]. In this study, tobacco was found to be a protective factor that could slow down the development of the most severe stages of MM, including CNV.

Over the past few years, the relationships between environmental factors, for example excessive near work and time spent outdoors, and the development of myopia have been studied [1]. In the current study, associations between these factors were investigated, but no statistically significant differences were found. According to these results, it is possible that these factors play a major role when comparing HM with emmetropic population, and have less importance between the subjects with and without MM. The subjective and retrospective nature of the questionnaire used to obtain information could explain the absence of significant differences between the groups. Therefore, more objective methods should be implemented in future studies, for example biomarkers of outdoor exposure such as vitamin D levels [44] or conjunctival ultraviolet autofluorescence [45–49].

Given that genetics have been shown to be important in the development of HM and MM in previous studies, the aim of this study was to verify the influence of genetics on MM development in two different cohorts of Spanish individuals. In a previous study, our group demonstrated that the *COL8A1* SNP rs13095226 was associated with an increase in axial length, and plays an important role in the development of CNV in highly myopic Caucasian patients [22]. The results of the current study reaffirm these findings in two cohorts, in which patients were classified according to a novel MM grading system. Also, the SNP rs669676 showed a close to significance protective effect with the development of MM. According to the literature, *COL8A1* encodes one of the two alpha chains of type VIII collagen. This protein regulates the activity of matrix metalloproteinases, so *COL8A1* may be involved in thinning and remodelling of the scleral extracellular matrix, which is known to increase the eyeball axial length [22]. The RPE and choroid are likely to be involved in this process, as it is thought that these changes occur via a molecular signalling cascade that involves the release of growth factors. These growth factors are transferred from the retina to the sclera, probably through the RPE and choroid [34]. This correlates with previous evidence that suggests that axial length has strong genetic components, and with the results of this study, in which patients in the CNV group exhibited increased axial length.

Several theories have been proposed to try to explain the relationship between *COL8A1* and the development of myopic CNV. Firstly, type VIII collagen is a component of Bruch’s membrane, therefore *COL8A1* may produce structural alterations in this membrane, which has been related with CNV formation. Secondly, the proteins encoded by *COL8A1* are involved in vascular endothelial growth factor-mediated endothelial cell migration during angiogenesis [22]. Based on these theories, we evaluated the expression of these genes in different ocular tissues from European cadaveric donors. mRNA analysis was performed, and the results showed that *CCDC102B*, *COL8A1*, and *SCO2* were all expressed in the retina, RPE, and sclera. Notably, the expression of *COL8A1* was 300 times greater in the sclera than in the retina. Thus, we showed that *COL8A1* is primarily expressed in the sclera, and that MM patients with CNV exhibit a higher frequency of the risk genotype and increased axial length. According to these data, it can be hypothesized that *COL8A1* may play an important role in anteroposterior axis elongation of the eye, which is known to contribute to the development of CNV in highly myopic patients. Several theories have been proposed to explain the development of myopic CNV [50,51], and the results of the current study correlated with both the heredodegenerative and mechanical theories.

*Chromosome 15q14* showed a significant association with MM, although this was lost after the Bonferroni correction was applied, and also an association close to significance in allele frequency between subjects with and without HM. Therefore, this gene could be related with the development of HM and MM, although it seems like in these cohorts does not have the same power as in Asian studies. Previous studies have shown an association between a locus in chromosome 15q14 and HM in Japanese populations [15]. This can be explained by the proximity of this locus to *GJD2* and *ACTC1*, two genes expressed in the retina that are thought to be involved in eye growth regulation [52]. Therefore further investigation is required to elucidate the contribution of this gene in the pathogenesis of HM and MM.

The rest of the studied genes did not show any associations with HM, MM or CNV, proving that the allele and genotype frequency values obtained were those expected for a European population. Despite this, these genes have been shown to play an important role in the development of HM and MM in previous studies. In a recent two-stage GWAS of MM, an association between a *CCDC102B* locus and MM was identified. In addition, the expression of this gene in the human retina and RPE-choroid has been confirmed, supporting the theory that it may promote atrophy of the RPE-choroid in subjects with MM [23]. *BLID* is thought to encode an inducer of apoptotic cell death which is known to play an important role in pathological myopia [14,21]. Furthermore, *BLID* has also been shown to be expressed in the human retina [21]. Another gene that has been associated with HM is *SCO2* [12,13,53]. *SCO2* deficiency may affect normal copper metabolism in ocular tissues, resulting in increased oxidative stress, altered retinal function, and ultimately HM and MM. Finally, *PAX6*, one of the most studied genes with respect to HM, was also analysed in this study. This gene is known to play an important role in the control of eye globe growth [18,19,54], and a suggestive association with HM was demonstrated in a meta-analysis of studies performed mainly in Chinese populations [20].

Nowadays, GWASs have made it possible to identify multiple SNPs and investigate their association with HM and MM. In the current study, we focused only on relevant SNPs previously associated with HM and MM in Asian populations, so a limited number of genes were analysed. Although this may be a limitation of the study, further analyses can be carried out at a later stage using the available database. On the other hand, a strength of this study is that all participants came from a similar ethnic background, which reduced the possibility of heterogeneity in the study population.

In conclusion, among the eight SNPs that were analysed in this study, the *COL8A1* SNP rs13095226, and the *chromosome 15q14* SNP rs634990 showed an association with MM in this Spanish population. This suggests that *SCO2*, *CCDC102B*, *PAX6*, and *BLID* do not play important roles in the development of this disease in Spanish population. On the other hand, additional factors that were studied, namely age, sex, smoking status, and pregnancy history, were found to be associated with MM and CNV in this Spanish population. In the future, more genetic and epigenetic studies should be performed to elucidate the importance of each of these factors in the development of HM and MM.

## Supporting information

S1 Data (XLSX)
